# FPGA-accelerated streaming data reduction achieving an average compression ratio over 8000 in a 17.4 kHz, 840 kpixel CITIUS detector for quasi-elastic gamma-ray scattering

**DOI:** 10.1107/S1600577526000883

**Published:** 2026-03-25

**Authors:** Haruki Nishino, Masashi Kobayashi, Toshiyuki Nishiyama Hiraki, Yoshiaki Honjo, Kyosuke Ozaki, Mitsuhiro Yamaga, Nobumoto Nagasawa, Yoshitaka Yoda, Yasumasa Joti, Makina Saito, Takaki Hatsui

**Affiliations:** a Japan Synchrotron Radiation Research Institute (JASRI) 1-1-1 Kouto, Sayo-cho Sayo-gun Hyogo679-5198 Japan; b RIKEN SPring-8 Center, 1-1-1 Kouto, Sayo-cho, Sayo-gun, Hyogo679-5148, Japan; c Department of Physics, Graduate School of Science Tohoku University Sendai Miyagi980-8578 Japan; Paul Scherrer Institut, Switzerland

**Keywords:** detectors, data reduction, quasi-elastic gamma-ray scattering, SPring-8, data acquisition, data analysis, field-programmable gate arrays, high-performance computing

## Abstract

We present a data-acquisition and -analysis framework for quasi-elastic gamma-ray scattering (QEGS) experiments at BL35XU of SPring-8, equipped with an 840 kpixel CITIUS X-ray detector operating at 17.4 kHz. The detector produces data at 27 GB s^−1^ (216 Gbps), and typical experiments involve acquisition over beam-time periods longer than 24 h, generating datasets of 2.3 PB per day. To handle this volume, we constructed a data-handling pipeline consisting of the detector, data reduction at the beamline and analysis tools at the data center. The data reduction employs field-programmable gate array (FPGA)-accelerated per-pixel processing to reduce data entropy, followed by Zstandard compression on CPUs, achieving an average compression ratio of over 8000. The compressed data are transferred to the SPring-8 data center within two to three minutes of data acquisition. At the data center, analysis tools are provided via the *Open OnDemand* platform, enabling incremental integration and spectral analysis through a web-based interface without the need for high-performance-computing command-line interaction. This data-handling pipeline has been applied in QEGS user experiments, where it enabled timely feedback on experimental data, with integrated results available within six minutes and spectral analysis within seven minutes of integration.

## Abstract 2

A high-throughput field-programmable gate array (FPGA)-accelerated data-reduction and -analysis pipeline combined with high-performance computing enables the continuous handling of a 216 Gbps data stream from quasi-elastic gamma-ray scattering experiments at SPring-8.

## 1. Introduction

Quasi-elastic gamma-ray scattering (QEGS) based on synchrotron radiation time-domain interferometry enables measurements of microscopic dynamics in reciprocal space over time windows from a few to several hundred nano­seconds (Saito *et al.*, 2017 ▸, 2012 ▸; Baron *et al.*, 1997 ▸). This technique has provided valuable microscopic insights into the properties and functions of diverse systems, including liquids, gases and soft matter (Nagao *et al.*, 2021 ▸; Saito *et al.*, 2022 ▸, 2025 ▸). However, time-domain QEGS measurements have so far been unable to access the sub-nanosecond regime because the time resolution is limited by avalanche photodiode detectors (Saito *et al.*, 2017 ▸). In contrast, sub-nanosecond dynamics can be probed using energy-domain QEGS measurements (Mitsui *et al.*, 2022 ▸). Nevertheless, precise measurements of wavevector dependence in the energy domain remain challenging, as they typically rely on point detectors such as NaI(Tl) scintillation detectors (Tischler *et al.*, 1996 ▸; Masuda *et al.*, 2009 ▸; Mitsui *et al.*, 2018 ▸, 2022 ▸).

**Figure 1:**
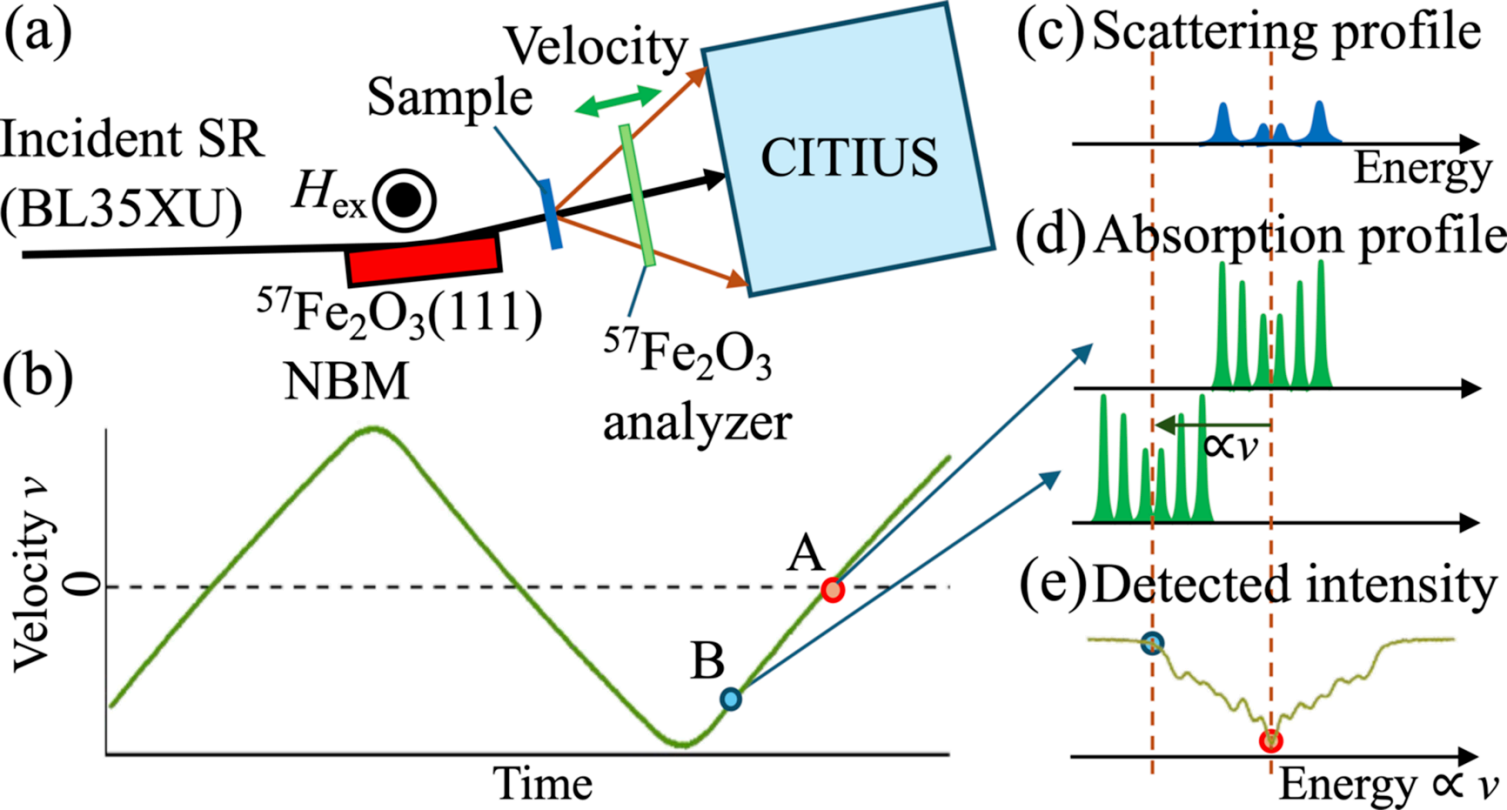
Principle of a QEGS experiment. (*a*) Schematic drawing of the experimental setup, where SR means synchrotron radiation. (*b*) Velocity profile applied to drive the ^57^Fe_2_O_3_ nuclear analyzer to induce the Doppler shift of the absorption energy. (*c*) Energy probability distribution of multiline gamma rays scattered from the sample. (*d*) Multiline absorption profiles of the analyzer at velocity points A and B in panel (*b*), shown with respect to the energies of the scattered gamma rays. (*e*) Gamma-ray intensity detected by CITIUS as a function of Doppler energy (proportional to Doppler velocity *v*).

Recently, we reported a new energy-domain QEGS using multiline gamma rays at SPring-8 BL35XU that can efficiently cover a wide time range from 100 ps to 100 ns (Saito *et al.*, 2024 ▸). Importantly, by employing the X-ray imaging detector CITIUS (Hatsui *et al.*, 2026 ▸), this system enables precise determination of wavevector-dependent dynamics. Here, we briefly explain why a high-frame-rate detector such as CITIUS, operating at 17.4 kHz, is required to access nano­second-scale dynamics. Fig. 1 ▸ shows (*a*) a schematic drawing of the experimental configuration and (*b*)–(*e*) the principle of energy analysis of quasi-elastic scattering. The ^57^Fe_2_O_3_ nuclear Bragg monochromator (NBM) produces gamma rays with a comb-like excitation spectrum consisting of multiple peaks with linewidths of several tens of neV, distributed over an energy range of several µeV around 14.4 keV. Gamma rays scattered by the sample are transmitted through an oscillating ^57^Fe_2_O_3_ nuclear analyzer, which selectively absorbs photons depending on their energies as determined by its Doppler velocity *v*.

By accumulating transmitted intensities corresponding to each analyzer velocity, the quasi-elastic energy spectrum can be reconstructed at each pixel of CITIUS, analogous to Mössbauer absorption spectroscopy. When the detector acquires images at a sufficiently high frame rate to resolve individual velocity points during the Doppler scan, each frame corresponds to a well defined analyzer velocity. Summing frames within each velocity bin enables reconstruction of diffraction intensities as a function of Doppler energy at every pixel, allowing simultaneous wavevector- and energy-resolved measurements. The typical analyzer frequency is around 10 Hz. To accurately reconstruct the multiline energy spectrum, transmitted intensities must be recorded at several hundred velocity points within each oscillation cycle. This requirement translates into a detector frame rate of the order of several kilohertz, which is fulfilled by CITIUS. Such high-resolution energy spectra allow measurements of quasi-elastic scattering broadening Γ spanning from neV to µeV, corresponding to microscopic relaxation times, τ ≃ ℏ/Γ, in the range from 100 ps to 100 ns.

At SPring-8 BL35XU, this QEGS scheme is implemented using an 840 kpixel CITIUS detector. The detector typically operates at a frame rate of 17.4 kHz and provides a nominal quantum efficiency as high as 81% at 14.4 keV, with a small pixel size of 72.6 µm^2^. The high frame rate and the large pixel number of CITIUS enable the acquisition of precise QEGS energy spectra with significantly higher wavenumber resolution compared with previous experiments using point detectors. While state-of-the-art photon-counting detectors reach frame rates exceeding 1 kHz (Donath *et al.*, 2023 ▸), CITIUS adopts a charge-integration approach, which enables reconstruction of photon energy information and suppression of background contributions from photons outside the energy range around 14.4 keV. Since its installation, the system has been used in more than 564 h of user experiments as of August 2025. Some scientific results were reported elsewhere (Moroboshi *et al.*, 2024 ▸).

The system employs an 840 kpixel CITIUS detector that produces data at 216 Gbps, which requires substantial resources for real-time data handling and long-term storage. During beam time, users need access to preprocessed detector data with a latency of about one second. To satisfy this requirement, the detector readout utilizes field-programmable gate array (FPGA)-accelerated per-pixel processing at the beamline’s edge. The duration of QEGS experiments is typically more than 24 h, generating datasets of 2.3 PB per day.

In this work, we present our approach to managing such large data volumes by combining FPGA-accelerated streaming data reduction with a high-performance computing (HPC) system, achieving an average compression ratio of over 8000.

## 2. CITIUS detector

Each CITIUS sensor has a pixel array of 384 × 728 pixels with a pixel size of 72.6 µm, corresponding to ∼280 kpixels. Each frame of data contains 384 × 768 pixels, including additional pixel circuit outputs without pixel connection. The sensors use 650 µm-thick silicon, providing a nominal quantum efficiency as high as 81% for 14.4 keV photons. The typical noise level is 40 e^−^ r.m.s., equivalent to 0.01 photons at 14.4 keV.

**Figure 2:**
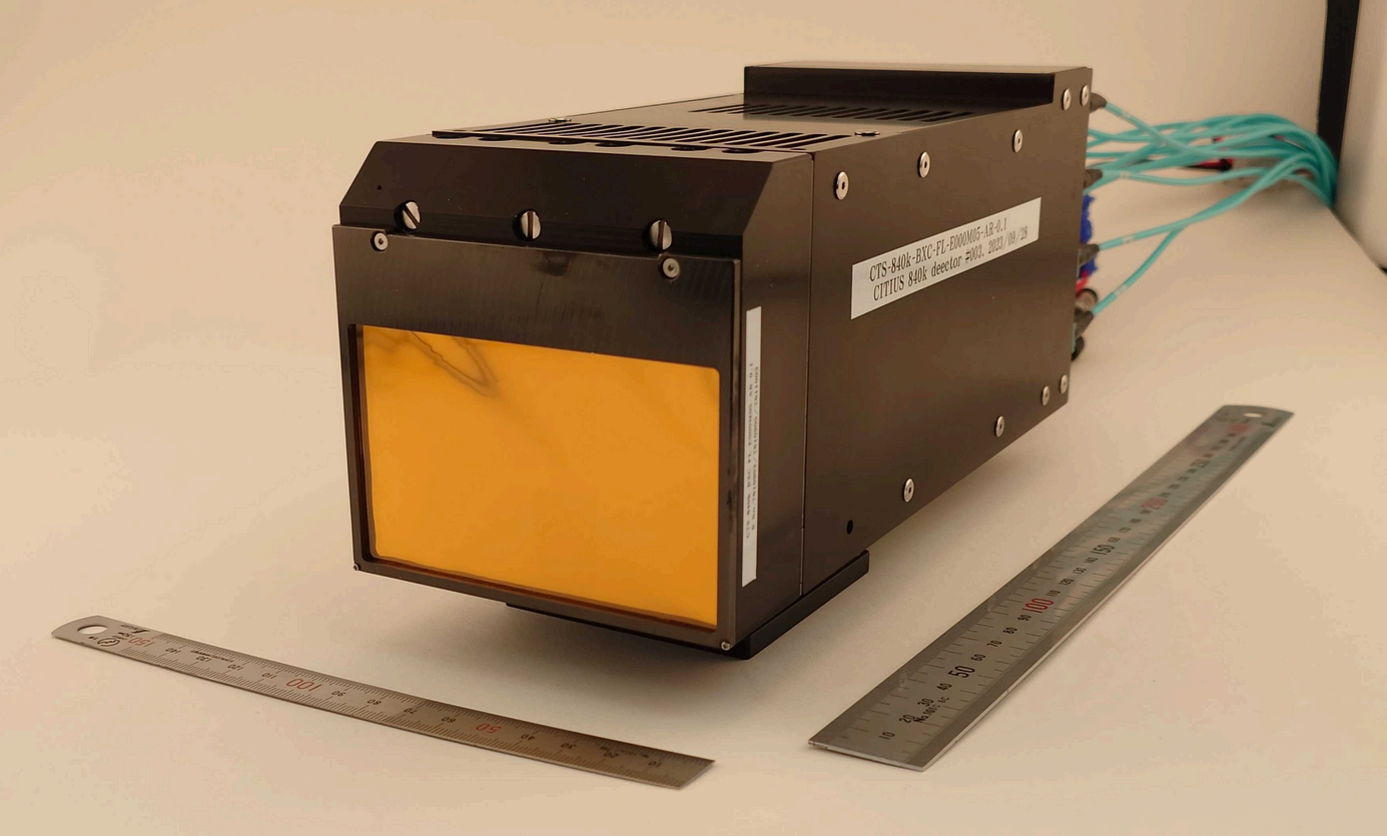
Photograph of the 840 kpixel CITIUS detector used in the QEGS experiments at BL35XU of SPring-8. The detector has an entrance window made of an aluminized polyimide film. Three sensors are integrated inside the housing to provide an imaging area of 89.3 mm × 52.9 mm. The 150 mm and 300 mm rulers indicate the physical dimensions of the detector module.

The QEGS measurement system employs a detector consisting of three CITIUS sensors, providing a total of 840 kpixels. The detector has an imaging area of 89.3 mm × 52.9 mm with 2.8 mm gaps between sensors. Fig. 2 ▸ shows a photograph of the detector system. Each sensor outputs data through 48 optical lanes, giving a total of 144 lanes. These lanes are connected via three multi-fiber push-on (MPO) connectors on the backside of the detector.

The sensors are driven by an externally supplied system clock derived from the accelerator radio frequency (RF), ensuring bunch synchronization with the SPring-8 storage ring (Nishino *et al.*, 2023 ▸). The detector operates at a frame rate of 17.4 kHz, and data are acquired in trains of consecutive frames, initiated by an external trigger. In typical QEGS experiments, each train contains 8184 frames, with a repetition rate of 2.12 Hz and an idle interval of eight frames between successive trains. The train trigger signal is also derived from the accelerator RF so that the trigger period exactly equals an integer multiple of the period of the nuclear analyzer. As a result, each frame index within a train corresponds to a specific analyzer velocity. By integrating trains at each frame index, diffraction images can be reconstructed as a function of nuclear-analyzer velocity, which is the basis of the train-integration scheme used in QEGS.

The detector was first deployed in the QEGS system at BL35XU of SPring-8 in July 2022. Since then, it has been used in user experiments for the collaborative development of the QEGS system. Following the commissioning of the SPring-8 data center in 2023, recorded datasets have been stored and analyzed there, supported by newly developed tools for automated QEGS data processing.

## 3. Data-reduction pipeline

### 3.1. Overview

**Figure 3:**
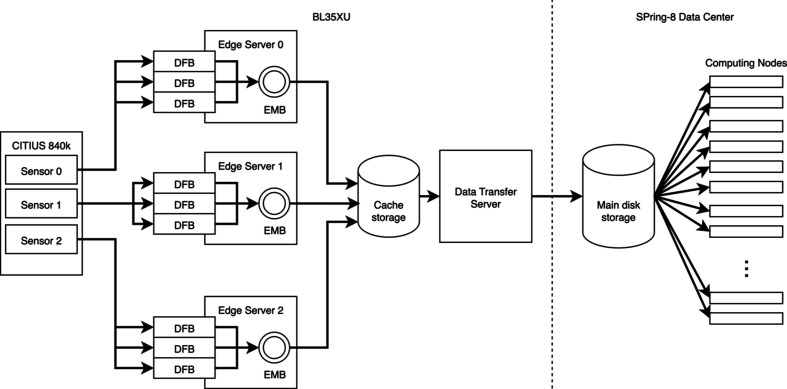
Data flow from the 840 kpixel CITIUS detector at BL35XU to the SPring-8 data center. Sensor data are processed by edge servers with DFBs, transferred via cache storage and a data-transfer server, and stored in the main disk storage in the data center.

Fig. 3 ▸ illustrates the data flow from the 840 kpixel CITIUS detector to the SPring-8 data center. The sensors transmit data using the scalable low-voltage signaling with embedded clock (SLVS-EC) protocol. Each pixel value is represented by 14 bits, including 2 bits for gain information. Per-pixel gain selection is performed at the sensors. Operating at a frame rate of 17.4 kHz, the 840 kpixel CITIUS detector generates a total throughput of 27 GB s^−1^. Table 1 ▸ summarizes the performance of the data-handling pipeline.

To handle this throughput, each sensor is connected to a dedicated edge server that receives, calibrates and stores the incoming data. Each edge server is equipped with three data-framing boards (DFBs), which perform the initial calibration of the sensor output. The edge servers write the compressed data to cache storage located at the beamline. A data-transfer server connected to this cache storage subsequently transfers the compressed data to the main disk storage of the data center. Users can then analyze the transferred data using the computing nodes available at the data center.

### 3.2. FPGA data handling

The DFB is a custom PCI Express board incorporating three Intel Arria 10 FPGAs, designed to receive and preprocess data streams from CITIUS sensors. Data from each sensor are transmitted via 48 SLVS-EC lanes, grouped into six physical-layer blocks (PHYs). Each edge server hosts three DFBs. On each DFB, two first-stage FPGAs are dedicated to data reception and per-pixel calibration, each handling two PHYs (16 lanes). The remaining second-stage FPGA, with larger logic capacity and access to on-board DRAM, performs subsequent processing and communication. Resource utilization for the first-stage FPGAs is 31% for adaptive logic modules (ALMs), 33% for M20K memory block and 30% for digital signal processing (DSP) blocks. For the second-stage FPGA, the utilization is 23% for ALMs, 51% for M20K and 7% for DSP blocks.

The DFB also contains on-board memory for storing per-pixel calibration parameters. The per-pixel processing steps include:

(1) Background subtraction using pre-calibrated offset values.

(2) Thresholding, where pixels below a predefined threshold are set to zero. The full image array is preserved while reducing entropy for compression.

(3) Frame summation, in which two consecutive frames are summed to produce a single output frame, resulting in an output frame rate of 8.7 kHz. Although the DFB supports summing an arbitrary number of consecutive frames, a summation factor of two was selected.

While the DFB firmware includes functionality for per-pixel gain calibration, this step is deferred to the post-analysis stage in this application. In typical QEGS experiments, the detector receives a total flux of the order of 10^4^ photons per second. At a 17.4 kHz frame rate, this corresponds to an order of one photon per frame per sensor and an occupancy of the order of 10^−6^ photons per pixel per frame. The thresholding and frame summation reduce the data entropy and increase compressibility for downstream processing.

After FPGA processing, the resulting data are stored in the on-board DRAM of the DFB. These data are stored in a cached binary format, consisting of a train header, followed by frame headers and the corresponding image data. Data in this format are then transferred via direct memory access (DMA) to the edge-server main buffer (EMB), which comprises eight 128 GB DDR4 DRAM (Samsung Electronics Co., Ltd., M393AAG40M32-CAE). This transfer occurs over PCI Express Gen3 ×8 interfaces. With three DFBs per edge server, the system utilizes a total of nine links. The DFB output corresponds to a summed frame rate of 8.7 kHz, represented in 32-bit floating-point format, yielding a total throughput of 31 GB s^−1^ for the three sensors combined. The two-frame summation reduces the data bandwidth below the achieved sustained data-transfer capacity of the DFB interfaces while maintaining a sufficiently high frame rate for the QEGS experiment.

### 3.3. Compressed data recording to the cache storage disk

A dedicated data acquisition (DAQ) program running on each edge server writes the data to cache storage implemented at BL35XU. During this process, on-the-fly compression is performed on the CPUs using the standard Zstandard algorithm (Collet & Kucherawy, 2021 ▸). The compression level is set to 1 to maximize throughput and no custom dictionaries are employed. The DAQ program spawns one processing thread per DFB. Each of these threads configures the Zstandard library to utilize two internal worker threads for parallel compression. Consequently, a total of six Zstandard worker threads run concurrently on each edge server. For a typical QEGS experiment, the compression load corresponds to an average utilization of 4.6 CPU cores, while the server is equipped with two CPUs (Intel Corporation, Xeon Gold 6252N) providing 48 physical cores in total. In previous QEGS experiments, compression ratios ranged from 1 × 10^3^ to 9 × 10^3^, with an average of 8.6 × 10^3^. This corresponds to an average write rate of 3.6 MB s^−1^ to the cache storage. This compression is essential to reduce data volume and enable efficient transfer and analysis at the data center.

The cache storage comprises 16 6.4 TB NVMe SSDs (KIOXIA Corporation, CM6) and is shared among the three edge servers via the *BeeGFS* (https://www.beegfs.io/c/) parallel file system. Each edge server connects to the cache storage through an InfiniBand HDR100 interface, with data transferred using remote DMA (RDMA). The SSDs are configured as two RAIDZ1 (RAIDZ1 is a ZFS-based single-parity RAID configuration, similar to RAID5) arrays, providing an effective storage capacity of 84 TB. This capacity is sufficient for typical QEGS experiments and also provides a margin to accommodate experiments with lower compression ratios.

### 3.4. Data transfer to SPring-8 data center

Data stored in the cache storage are transferred to the data center. The data-transfer server, connected to the cache storage via InfiniBand HDR100, runs a dedicated transfer tool that monitors the cache storage for new files, calculates checksums and initiates transfers to the main disk storage of the data center. Data transfer proceeds concurrently with the detector’s ongoing recording to the cache storage. Newly acquired files typically reach the data center within two to three minutes of DAQ. The network bandwidth between the data-transfer server and the data center is 10 Gbps (as of August 2025), exceeding the compressed data rate of 3.6 MB s^−1^.

## 4. Data-analysis tool at SPring-8 data center

**Figure 4:**
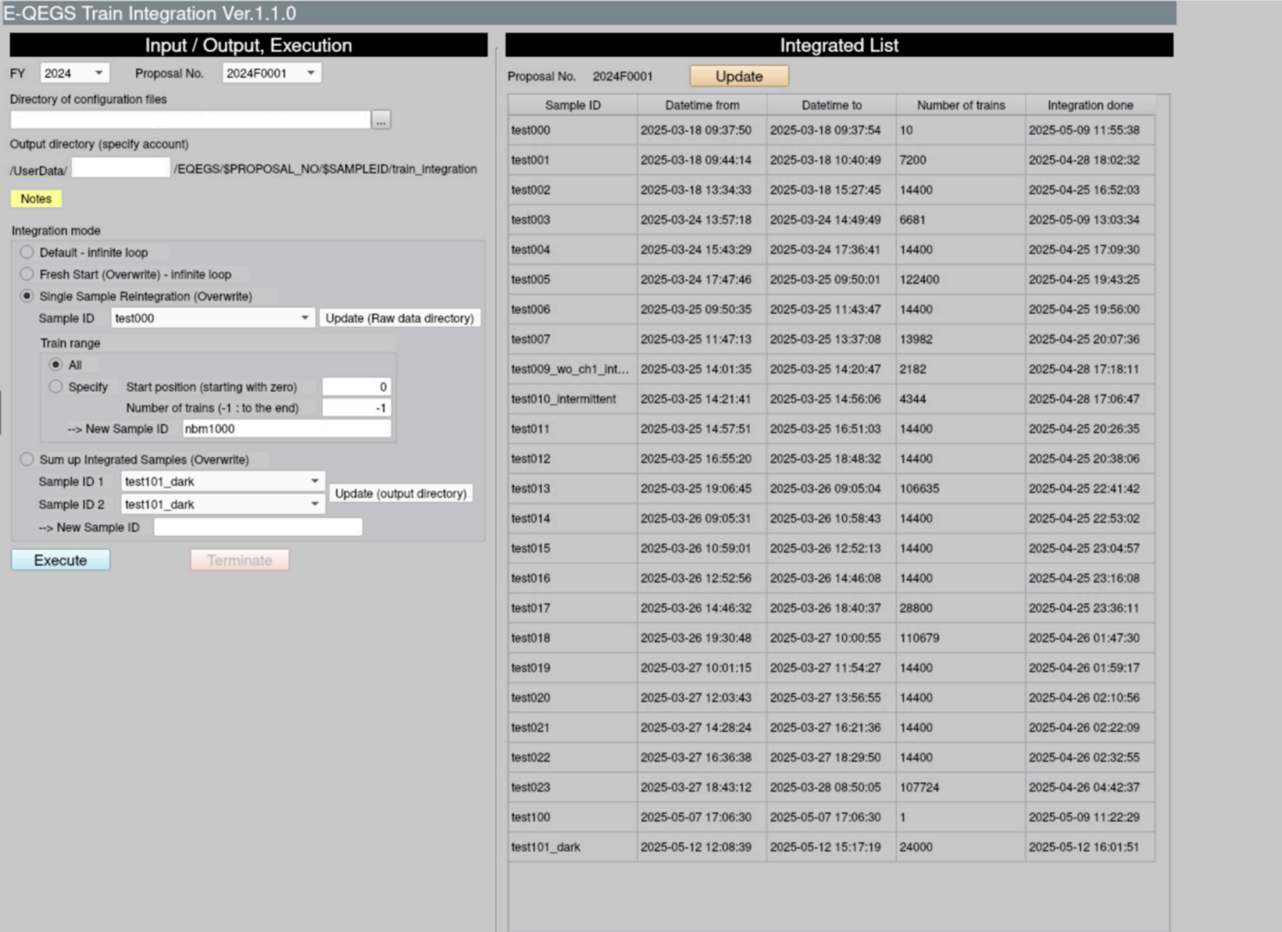
Graphical user interface for the train-integration tool developed for QEGS experiments. The panel on the left shows input and execution settings, including configuration files and integration modes. The panel on the right displays the integrated list of samples, with metadata such as sample ID, acquisition period, number of trains and integration status. Personal information has been masked in this figure.

While the compressed data stream averages 3.6 MB s^−1^, decompression and memory mapping require the analysis to sustain the equivalent uncompressed rate of 31 GB s^−1^ in memory. Achieving such performance demands parallelization using an HPC system. This technical requirement can pose a challenge for new users. To address this, we developed a suite of data-analysis tools for QEGS experiments using CITIUS at the SPring-8 data center. Since HPC environments often demand specialized knowledge, we adopted *Open OnDemand* (Hudak *et al.*, 2018 ▸) to provide an accessible graphical interface. These tools can be launched from a web browser, allowing analyses without interaction with command-line HPC environments, as shown in Figs. 4 ▸ and 5 ▸.

The first step in the QEGS data-analysis workflow is train-wise integration. As described in Section 2, CITIUS is synchronized with the accelerator RF and the nuclear analyzer is locked to the same reference. By integrating signal intensities over long periods for each frame index, the signal-to-noise ratio is improved. The output from this step consists of integrated images for each of the three sensors corresponding to every frame index. The integration tool automatically detects newly transferred data files, performs incremental integration and updates results. The data are divided into sub-datasets for parallelization and the tool achieves a throughput equivalent to processing 88 kframes s^−1^ for the 840 kpixel CITIUS detector. It runs concurrently with DAQ, and integrated results are typically available within six minutes, including data-transfer latency.

In addition to the integration tool, we developed a spectrum-analysis tool to provide users with a measured intermediate scattering function. This tool enables spectral analysis of QEGS data via a graphical user interface on the *Open OnDemand* platform. The analysis proceeds in three sub-processes: (1) reconstruction of the diffraction image, (2) computation of wavevector-dependent scattering intensity and (3) evaluation of the intermediate scattering function.

The first process merges train-integrated images from the three sensors into single diffraction intensity images at each frame index, applying geometrical corrections. The second process computes the scattering intensity as a function of the wavevector for all frame indices. This process involves mapping all the pixels to wavenumber space for every frame index, making it the most computationally intensive part of the analysis pipeline. To address this, the process utilizes parallelization across multiple computing nodes by dividing a train into multiple chunks of frame indices. As explained in the *Introduction*[Sec sec1], the frame index corresponds to the Doppler energy shift. Using the wavevector-dependent scattering intensity from the second process, the third process converts the intensities into absorption energy spectra for a given wavenumber range. Finally, the intermediate scattering function is obtained by performing a Fourier transformation on the energy spectra to visualize the relaxation shape in time domain. A quasi-elastic energy spectrum and the corresponding intermediate scattering function can be obtained within seven minutes of train integration. The interface provides intuitive controls for data selection, visualization and parameter tuning, enabling prompt feedback during or after experiments.

## 5. Discussion

This work demonstrates that QEGS experiments using a detector with a sustained data output rate of 216 Gbps can be operated continuously by an integrated pipeline combining FPGA-assisted per-pixel processing, CPU-based lossless compression and HPC-assisted analysis. This system enables feedback during beam time, allowing evaluation of the intermediate scattering function. Achieving rapid feedback required an analysis framework that is accessible to a broad range of users, so that data analysis can proceed without waiting for dedicated support from experts.

Browser-based analysis environments for general-purpose use, such as *JupyterHub* (Jupyter, 2018 ▸) and *OpenOnDemand*, have been introduced at photon science facilities (*e.g.* Bard *et al.*, 2022 ▸; Dimper *et al.*, 2019 ▸). These platforms provide convenient access to HPC resources, but effective use of HPC parallelization requires user expertise, such as selecting appropriate parallelization parameters, configuring job-scheduler settings, and managing data partitioning and I/O. In this work, *Open OnDemand* is used as a platform to provide analysis applications developed for QEGS experiments, in which the parallel execution, job submission and resource configuration are predefined within the tools. As a result, users can run the analysis workflow through a web interface while sustaining a data throughput that exceeds the data rate of the CITIUS detector.

The current system has a bandwidth limitation from the DFBs to the EMBs. The bandwidth is currently constrained by the single PCI Express Gen3 ×8 interface on the DFB cards, which necessitated two-frame summation (reducing the effective frame rate to 8.7 kHz) to enable continuous recording. This bottleneck can be addressed in future upgrades by enabling the second PCI Express Gen3 ×8 interface available on the DFB, which would support the 17.4 kHz readout without summation and thereby improve the energy resolution of QEGS measurements. Furthermore, as indicated by the low photon occupancy of 10^−6^, there remains potential for further data reduction. A next version of the firmware will provide the capability to perform event detection and spectral reconstruction directly on the DFBs (Ozaki *et al.*, 2025 ▸). This approach is expected to improve the compression ratio and eliminate the computational cost of decompression, further accelerating the analysis pipeline.

## 6. Summary

We deployed an 840 kpixel CITIUS detector for the QEGS measurement system at BL35XU of SPring-8. The system produces data at 27 GB s^−1^ (216 Gbps) with a frame rate of 17.4 kHz. A typical QEGS beam time generates 2.3 PB per day. Handling such volumes exceeds the capabilities of the beamline computing resources, necessitating the implementation of an efficient data-reduction and -transfer pipeline.

To address this, we developed an FPGA-accelerated data-reduction pipeline using DFBs. The DFBs conduct calibration and per-pixel thresholding, as well as frame summation (two frames into one). This achieves an average compression ratio of over 8000 for the entire data-reduction pipeline. In a typical week-long QEGS experiment, data size was reduced from 19 PB to 2.2 TB. This enables compressed data to be transferred to the SPring-8 data center with a typical latency of two to three minutes.

At the data center, we implemented *Open OnDemand* to provide user access via web browsers. Transferred data are monitored by the train-integration tool, which performs incremental integration and updates results within six minutes. Using the spectrum-analysis tool at the data center, users at the beamline can compute the intermediate scattering function within seven minutes. This integrated framework for detection, data reduction and analysis has already been applied in user experiments, and has demonstrated its effectiveness in QEGS studies.

## Figures and Tables

**Figure 5:**
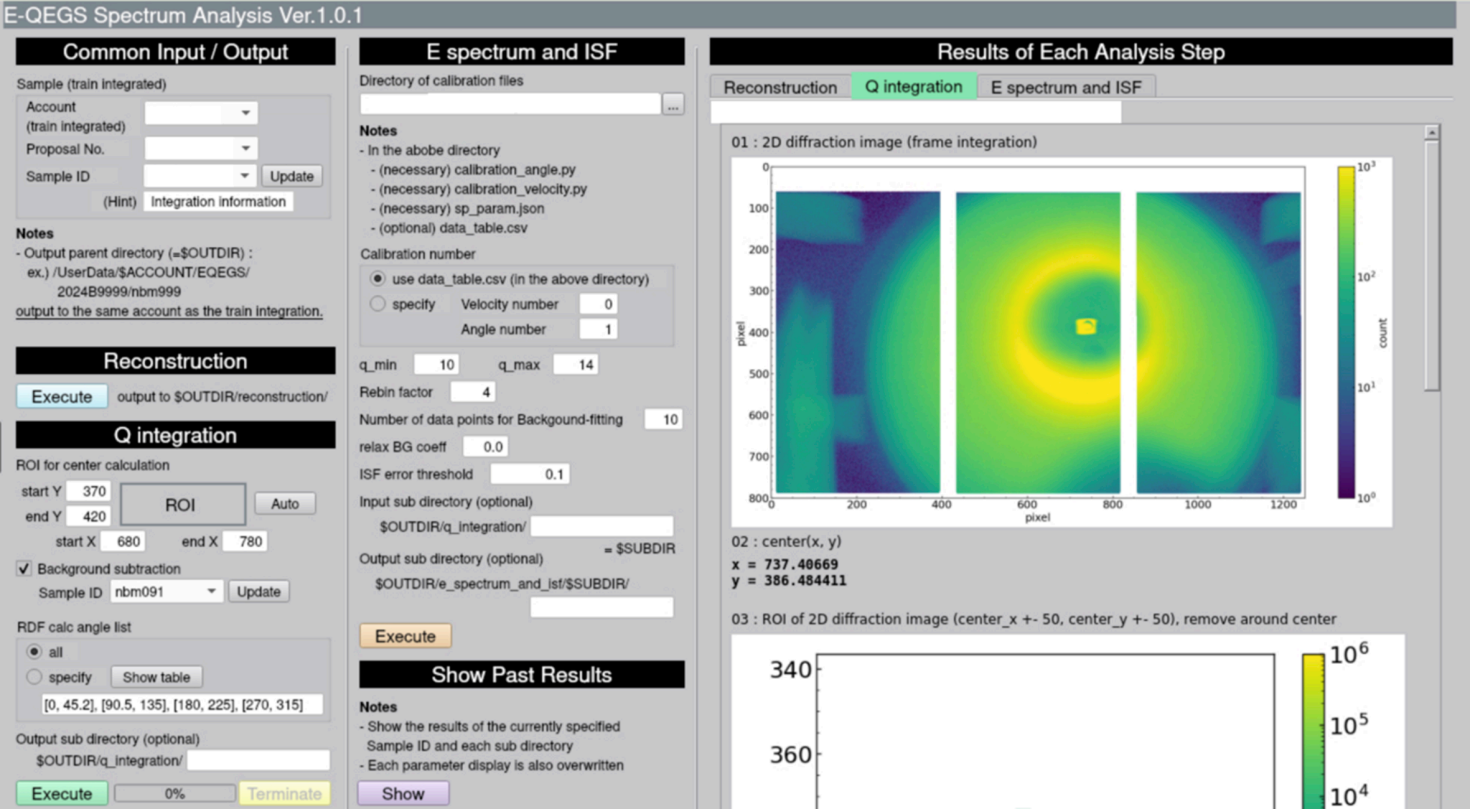
Graphical user interface for the spectrum-analysis tool developed for QEGS experiments. The left panel provides input/output settings and analysis parameters, while the central panel manages spectrum and intermediate scattering function computations. The right panel shows representative outputs from each analysis step, including a reconstructed two-dimensional diffraction image. Personal information has been masked in this figure.

**Table 1:** Performance summary of the CITIUS data-handling pipeline for QEGS experiments at BL35XU of SPring-8

Parameter		Values/expressions				Unit
Data-transfer section		Sensor → DFB	DFB → EMB	EMB → cache storage	Cache storage → main disk storage of the data center	N/A†
Straight-line distance		0	0	0	0.4	km
Interface	Physical layer	144-lane MPO optical fibers at 2.88 Gbps per lane ‡	PCIe Gen3 ×8, 9 links	InfiniBand HDR100, 3 links	10 GbE	N/A
	Logical layer	SLVS-EC v1.2	PCIe DMA	*BeeGFS* over RDMA	Rsync over TCP/IP	N/A
Frame rate		17.4	8.7§	8.7	8.7	kHz
Bit depth		14	32	32	32	Bit per pixel
Data representation		12-bit ADC value + 2-bit gain flag	Floating point	Floating point	Floating point	N/A
Compression		None	None	Zstandard	Zstandard	N/A
Data format		Serialized stream	Cached binary	Cached binary	Cached binary	N/A
Average throughput during deployment¶		27	31	3.6 × 10^−3^	3.6 × 10^−3^	GB s^−1^
		2.3	2.7	3.1 × 10^−4^	3.1 × 10^−4^	PB day^−1^
Average compression ratio¶		1	1	8.6 × 10^3^	8.6 × 10^3^	N/A
Cumulative data volume¶		55	62	6.8 × 10^−3^	6.8 × 10^−3^	PB

† Not applicable.

‡ Each sensor provides 48 SLVS-EC lanes.

§ Two consecutive frames are summed into one output frame.

¶ Average or cumulative values over 564 h of operation.

## Data Availability

Data will be available on request.
